# A Time Synchronization Protocol for Barrage Relay Networks

**DOI:** 10.3390/s23052447

**Published:** 2023-02-22

**Authors:** Woong Son, Jungwook Choi, Soobum Park, Howon Lee, Bang Chul Jung

**Affiliations:** 1 Department of Electronics Engineering, Chungnam National University, Daejeon 34134, Republic of Korea; 2 LIG Nex1, Yongin 16911, Republic of Korea; 3 School of Electronic and Electrical Engineering and IITC, Hankyong National University, Anseong 17579, Republic of Korea

**Keywords:** time-division multiple access (TDMA), wireless multi-hop ad hoc network, barrage relay network, time synchronization, network time reference selection, cooperative transmission

## Abstract

Time-division multiple access (TDMA)-based medium access control (MAC) protocol has been widely used for avoiding access conflicts in wireless multi-hop ad hoc networks, where the time synchronization among wireless nodes is essential. In this paper, we propose a novel time synchronization protocol for TDMA-based cooperative multi-hop wireless ad hoc networks, which are also called *barrage relay networks (BRNs)*. The proposed time synchronization protocol is based on cooperative relay transmissions to send time synchronization messages. We also propose a network time reference (NTR) selection technique for improving the convergence time and average time error. In the proposed NTR selection technique, each node overhears the user identifier (UID) of other nodes, hop count (HC) from them to itself, and network degree, which denotes the number of 1-hop neighbor nodes. Then, the node with the minimum HC from all other nodes is selected as the NTR node. If there are multiple nodes with the minimum HC, the node with the larger degree is selected as the NTR node. To the best of our knowledge, the proposed time synchronization protocol with the NTR selection is introduced for the first time for cooperative (barrage) relay networks in this paper. Through computer simulations, we validate the proposed time synchronization protocol in terms of the average time error under various practical network scenarios. Furthermore, we also compare the performance of the proposed protocol with the conventional time synchronization methods. It is shown that the proposed protocol significantly outperforms the conventional methods in terms of the average time error and convergence time. The proposed protocol is shown to be more robust against packet loss as well.

## 1. Introduction

Time synchronization is an important component for avoiding access conflicts in time-division multiple access (TDMA) wireless networks. Maintaining sharp and precise time synchronization between communication devices can improve the data-rate and spectral-efficiency [1,2,3,4]. However, time synchronization in wireless ad hoc networks is very difficult because there is no control device such as an access point (AP) in wireless sensor networks. Therefore, the importance of time synchronization in a wireless distributed communication system such as a TDMA-based wireless ad hoc network can not be overemphasized.

Unfortunately, *clock drift*, which is defined as the time difference between nodes, occurs due to a hardware problem caused by voltage, aging, temperature, and thermal noise variation, etc. [5,6]. Even if a high-cost clock oscillator (CO) is used to minimize the clock drift, the clock drift can not be perfectly prevented even with a CO produced by the same manufacturing process at the same factory due to non-ideal clock rates between COs. Thus, physical clocks run at *different clock rates* between the same COs, and clock drift occurs according to the elapsed time. *Time synchronization* is to minimize clock drift so that nodes can maintain TDMA frames or time-slots according to their hardware clock progress.

To solve this problem, network time synchronization is divided into *out-of-band* and *in-band* time synchronization methods in general. In *out-of-band* time synchronization, a device can time-synchronize by receiving a time synchronization message including global time information from a separate clock source such as global navigation satellite system (GNSS) or global positioning system (GPS) [7,8]. This solution has the strong advantage that very precise time synchronization of a wide-area can be achieved; however, a GNSS or GPS receiver is too expensive to be equipped at a node. Moreover, the critical drawback of GNSS or GPS is that they are not available in non-line-of-sight (NLOS) environments such as tunnel, forest, and indoors.On the other hand, in *in-band* time synchronization, a device can time-synchronize by sharing a time synchronization message including their local time. Therefore, clocks of devices synchronize with the average clock of all of them in the same networks without any network time reference (NTR) nodes, or synchronize with the local clock of a NTR node.

For wireless ad hoc networks without a NTR node, time synchronization using the IEEE 802.11 time synchronization function (TSF) [9] is well-known as the most promising technique that can perform time synchronization in a distributed manner without a NTR node in wireless ad hoc networks. Although it is a non-optimal technique, it is still considered for wireless ad hoc networks because it has customers around the world. Time synchronization based on IEEE 802.11 TSF is well-known for its good scalability [10,11,12,13]. In addition, enhanced time synchronization techniques based on IEEE 802.11 TSF are still being studied [14,15,16,17,18,19,20,21,22,23,24]. However, even if time synchronization based on IEEE 802.11 TSF is used in multi-hop wiress ad hoc networks, there is a problem in that the time synchronization accuracy decreases as the hop distance increases [23]. On the other hand, several studies are related to time synchronization for TDMA-based wireless ad hoc networks with a NTR node [25,26,27,28] in Table 1.

In [25], they considered a TDMA-based multi-hop wireless ad hoc network with ad hoc nodes deployed in a 5×7 grid. They fixed a center node as a NTR node, and when the existing NTR node can not operate, a method of selecting a new NTR node by a neighbor node of the previous NTR node was proposed; however, this method cannot autonomously select an NTR node. In [27], they considered *k*-medoids clustering, which elects the central node as a NTR node, for selecting NTR nodes in TDMA-based vehicular ad hoc networks (VANETs). In [26,28], they considered only TDMA-based single-hop wireless ad hoc networks. Existing studies considering TDMA-based multi-hop wireless ad hoc networks [25,26,27,28] cannot propose a feasible technique for selecting NTR nodes in real-world scenarios.Therefore, as with the IEEE 802.11 TSF, even in the presence of a NTR node, the problem of time synchronization accuracy decreasing according to the hop distance still remains.

Meanwhile, the time slot structure for cooperative broadcasting is introduced for tactical mobile mesh networks at the physical-layer [29]. It is a technique to receive messages previously broadcasted from parent nodes in a time slot, and relay it to their child nodes in decode-and-forward manner. This technique is named as *barrage relay transmission* [30], and related studies are being conducted [31,32,33,34,35,36,37,38]. However, these studies assumed that all nodes are coarsely synchronized based on GNSS or GPS signals. In particular, in an environment where GNSS or GPS are not available, it is difficult to use barrage relay transmission. Moreover, it is also difficult to apply even to TDMA using short time slots or long packets such as narrowband systems. These critical problems will intensify as communication technology advances.

In this paper, we propose a novel time synchronization technique based on a cooperative barrage relay from a unique NTR node to all nodes for TDMA multi-hop wireless ad hoc networks. Using the proposed time synchronization technique, it is efficient and simple because it can time-synchronize all nodes with a small number of hops. We also propose a novel NTR selection technique using network information that can be scanned from other nodes. Using the proposed NTR selection technique, it is possible to select a unique NTR node that can minimize the number of hops required for relaying time synchronization messages to all nodes. Furthermore, it can improve robustness under packet loss scenarios in terms of time errors.

The outline of paper is composed as follows. The clock model and measurements are explained in Section 2. The overall procedure of the proposed technique is explained in Section 3. The simulation results are analyzed in Section 4. Finally, the conclusion is summarized in Section 5.

## 2. Clock Model and Measurement

**Figure 1:**
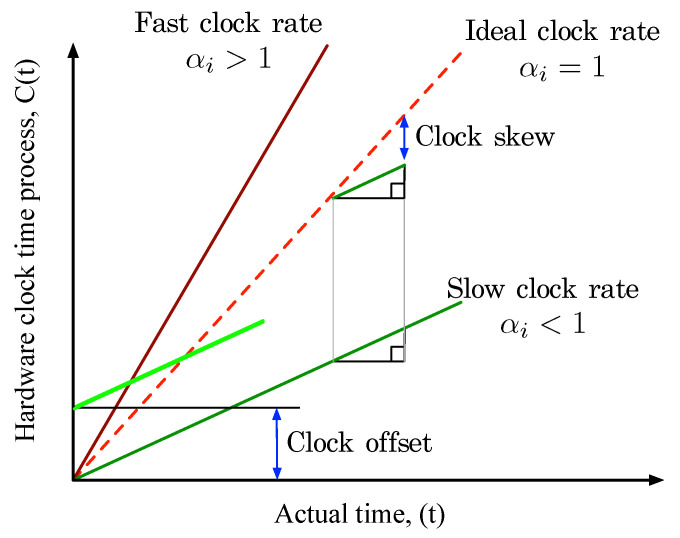
Clock model.

A precise time measurement is needed for communicating between devices in TDMA communication systems. So, a CO provides local clock to communication device (e.g., ad hoc or sensor node). Multiple communication devices communicate with each other while maintaining an imperfect clock. Therefore, they should be time-synchronized for reducing clock drift between communication devices as much as possible. Based on the traditional clock model [1,5,39,40], the clock model $C_{i}\left(t\right)$ of a node i∈{1,2,3,⋯,N}, which is known as the local time of node *i* for a wireless ad hoc networks consisting of *N* ad hoc nodes, when the actual time *t* is as follows.

(1) $$C_{i}\left(t\right)=\alpha_{i}t+\gamma_{i}\left(t\right)+\beta_{i},$$

where the $\alpha_{i}$ is the clock rate of a node *i*, the $\gamma_{i}\left(t\right)$ is a random process for modeling jitter as well as noise effect, and the $\beta_{i}$ is the clock offset. Most of literature papers for time synchronization use the term *clock skew*, which is defined as the *difference* between clock rates [1,39] as shown in Figure 1, and clock skew is used interchangeably with clock drift [40]. In particular, computing devices are equipped with a hardware clock-oscillator provided computer clock, which implements an approximation C(t) of real time *t*. In the perfect clock scenario, dC(t)/dt is equal to 1. However, a clock-oscillator is not an ideal hardware in the real world. Thus, clock-oscillator frequency will change instantaneously and unpredictably due to physical defects such as voltage, humidity, temperature, etc. When a node 1 and a node 2 have, respectively, clock rates $C_{1}\left(t\right)$ and $C_{2}\left(t\right)$ at real time *t*, $C_{1}\left(t\right)$ is not exactly equal to $C_{2}\left(t\right)$ due to physical defects. In addition, when the actual clock rate is equal to 1, the clock rate of a node *i* is expressed by $\alpha_{i}=1\pm \delta_{i}$ where the $\delta_{i}$ is the clock skew of a node *i*. It is also assumed that the clock rate does not change (constant clock rate assumption). Moreover, we assume that the $\gamma_{i}\left(t\right)=0$ for considering only the initial clock drift excepting jitter and noise effect.

When the actual time is equal to t=T (elapsed time without time synchronization), the clock difference $▵C_{i,j}\left(T\right)$ between the two nodes *i* and *j* can be expressed as

(2) $$▵C_{i,j}\left(T\right)=|C_{i}\left(T\right)-C_{j}\left(T\right)|=|\left(\alpha_{i}-\alpha_{j}\right)T+\left(\beta_{i}-\beta_{j}\right)|.$$

Furthermore, under a multiple *N* ad hoc nodes scenario with a unique NTR node *k*, the clock difference $▵C_{k}\left(T\right)$ between the NTR node and the entire nodes can be also expressed as

(3) $$▵C_{k}\left(T\right)=\left|C_{k}\left(T\right)-C_{\mathrm{avg}}\left(T\right)\right|=|\left(\alpha_{k}T+\beta_{k}\right)-\frac{1}{N}\sum_{i=1}^{N}\left(\alpha_{i}T+\beta_{i}\right)|.$$

In this paper, we define the clock difference $▵C_{k}\left(T\right)$ as the *average time error*. A node receiving a time synchronization message stores its local time based on the timestamp field in a time synchronization message. After that, the node increases the clock based on its own unique clock rate.

## 3. Proposed Time Synchronization

**Figure 2:**
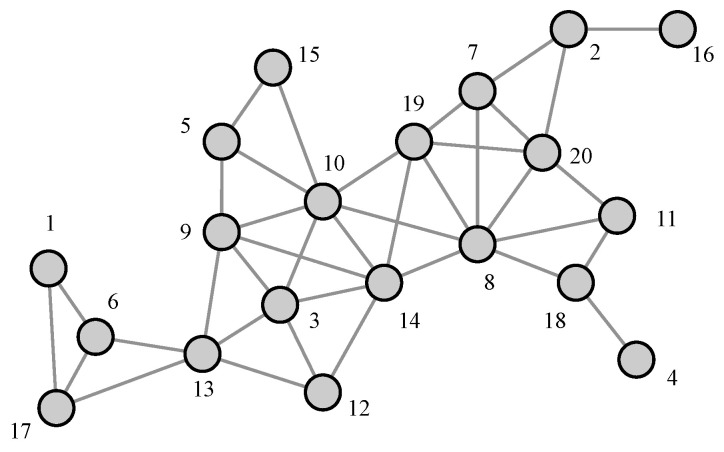
An example of network topology with 20 ad hoc nodes.

**Figure 3:**
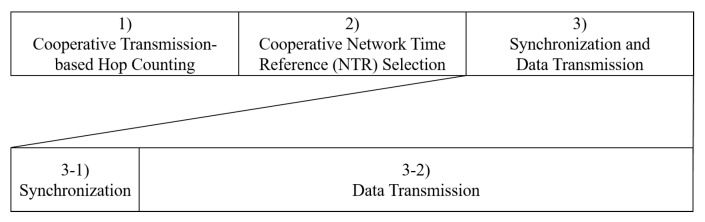
Three steps of the proposed time synchronization technique.

We describe our proposed time synchronization technique with the proposed NTR node selection in TDMA multi-hop ad hoc networks. Figure 2 shows an example of wireless ad hoc network topology consisting of fully-connected 20 ad hoc nodes. We assume that all nodes are coarsely-synchronized with each other after network configuration (slot-level synchronization assumption). We also assume that all nodes know other nodes’ user identifier (UID). Now, we explain the overall procedure of the proposed time synchronization technique consisting of three steps in Figure 3: (1) *cooperative transmission-based hop counting*, (2) *cooperative network time reference (NTR) selection*, and (3) *synchronization and data transmission*. In particular, the first and second steps should be performed at least once before the third step for acquiring and sharing information between nodes.

### 3.1. Cooperative Transmission-Based Hop Counting

**Figure 4:**
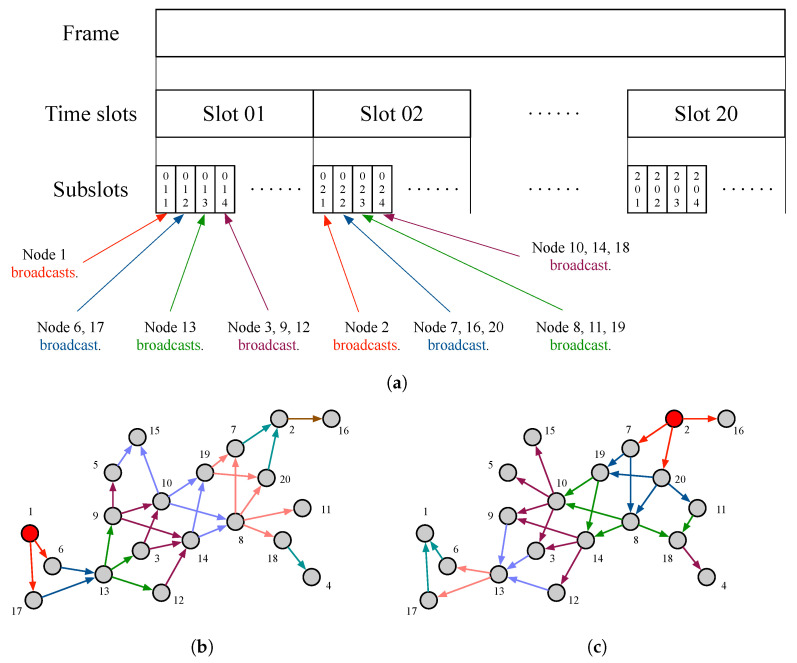
The basic concept of the proposed time synchronization technique based on cooperative barrage relay transmission. (**a**) Frame structure for time synchronizing in wireless ad hoc networks. (**b**) Cooperative barrage relaying a message from an assumed NTR node 1 via 8-hops for all existing nodes. (**c**) Cooperative barrage relaying a message from assumed NTR node 2 via 7-hops for all existing nodes.

In the *cooperative transmission-based hop counting* period, all nodes scan the *hop count (HC)* for all nodes and their *degree* (number of 1-hop neighbor nodes). Figure 4a shows the TDMA frame structure for cooperative transmission-based hop counting period for the proposed time synchronization technique. A unit TDMA frame consists of multiple time slots such as the number of nodes, and a unit time slot consists of multiple subslots for relaying messages. The 1st subslot in each time-slot is allocated only one node. We define this allocated node as an *assumed NTR node* for every time slot. Each time slot is for only one assumed NTR node, and a message is broadcasted from the assumed NTR node in the 1st subslot of each time slot. This message from the assumed NTR node in each time-slot include only assumed NTR node’s UID and HC field from the assumed NTR node. The role of this message is relayed from the assumed NTR node so that the receiving nodes can store the assumed NTR node’s UID and HC from the assumed NTR node. When nodes receive this message in which only the HC field is equal to 1, it can also be used to determined the degree. In addition, nodes received messages from the assumed NTR node broadcast before in the previous subslot, increasing only the HC field.Therefore, when multiple nodes transmit these messages within the same subslot, no collision occurs due to *barrage relay transmission* as shown in Figure 4b,c. In the 2nd subslot in each time-slot, 1-hop neighbor nodes from the assumed NTR node cooperatively relay that only the HC field increased 1 and maintain the UID of the assumed NTR node in each time-slot in the previously received message. Since the nodes receive these same messages in the 3rd subslot, there is no collision. When the cooperative transmission-based hop counting period is finished, all nodes can store the UID of other nodes and corresponding HC as well as their own degree.

### 3.2. Cooperative Network Time Reference (NTR) Selection

In the *conventional network time reference (NTR) selection*, the node with the smallest or the largest UID is selected as the NTR. Thus, if a node existing at the end of given wireless ad hoc network is selected as the NTR node for considering worst scenario in the conventional UID-based NTR selection, time synchronization error increases due to multi-hop relay and wireless resource is required due to time synchronization message relaying. However, a node relatively close to the center of given wireless ad hoc network that can relay the time synchronization message to all nodes constituting the network with the minimum hop can be selected as the NTR by applying the proposed NTR selection.

In the *cooperative network time reference (NTR) selection* period, each node shares information (UID, maximum HC, degree) stored in previous steps with other nodes first. The maximum HC indicates the largest HC among HCs to all nodes excepting itself. The procedure of information sharing between all nodes is also carried out in the same frame structure of the cooperative transmission-based hop counting period in Figure 4. However, the message includes only the UID of the assumed NTR node and the maximum HC among multiple HCs up to other nodes excepting the assumed NTR node. Since nodes receiving these messages in the each subslot perform cooperative relaying while maintaining the HC of the assumed NTR node, multiple identical messages can be relayed in the same subslot, so there is no collision.

**Figure 5:**
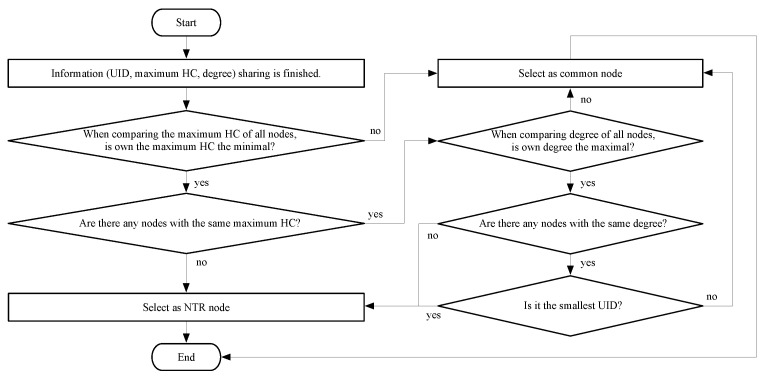
Flowchart of the proposed NTR selection.

At the end of the cooperative NTR selection period, only one NTR node can be determined by the proposed NTR selection algorithm in Figure 5. The selected NTR node is the node with the smallest HC among maximum HCs for all nodes, the largest degree and the smallest UID, the other nodes become common nodes. The NTR node selected in this algorithm can time-synchronize all nodes with the minimum number of hops, and a node that can reduce the time synchronization error by maximizing the number of children of the NTR node is selected.

### 3.3. Synchronization and Data Transmission

**Figure 6:**
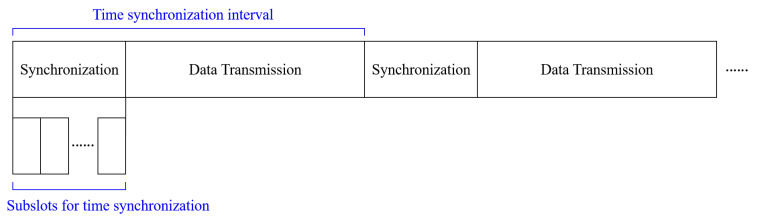
Synchronization and data transmission.

As shown in Figure 6, in the *synchronization and data transmission* period, all nodes can be synchronized via time synchronization message relaying, and transmit data via multi-hop relaying. With the NTR selection in the previous step, it was selected so that the time synchronization message can be relayed to all nodes with the minimum number of hop counts. Therefore, a number of subslots for time synchronization is required as much as the minumum number of hop counts of the selected NTR node. The time synchronization message includes the UID and timestamp of the NTR node. In addition, since the node receiving this message already knows the length of the subslot, it relays by only increasing the timestamp as much as the length of the subslot when relaying in the next subslot. Thus, these time synchronization messages from multiple nodes can be cooperative relayed, so these messages do not collide. Moreover, in a case of a child node with a single parent node, time synchronization is impossible if packet loss occurs. However, in a case of a child node with multiple parent nodes, time synchronization is possible if only one time synchronization message can be received even if packet loss occurs.

## 4. Simulation Results

**Figure 7:**
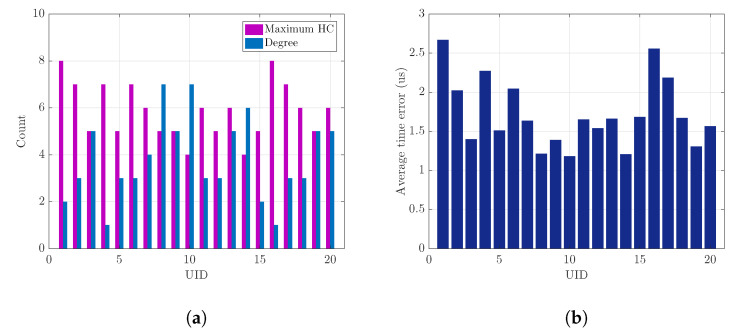
One snap-shot of wireless ad hoc network. (**a**) Maximum HC and degree. (**b**) Average time error.

We evaluate the performance of the proposed time synchronization technique with four NTR selection methods under various system parameters as summarized in Table 2. First, we consider one snap-shot of a wireless ad hoc network. In fact, Figure 2 is shown as a wireless ad hoc network consisting of 20 nodes within the size of area 1000 [m] ×1000 [m]. We assume that all nodes are fully-connected with the transmission range of each node being equal to 100 [m]. Moreover, we also assume that the clock rate of node *i* is an uniformly-distributed random variable $\alpha_{i}$ from the range $\left[1-\delta_{i},\;1+\delta_{i}\right]$, where the clock skew of node *i* is $\delta_{i}=0.0001$ following the IEEE 802.11 specification [9] for all *i*. Furthermore, we consider that 100 clock rates and initial clock offsets via a uniform-random manner. In addition, the time synchronization interval is assumed to be 0.1 [s]. Figure 7a is shown as the maximum HC and degree according to UIDs. As a result of performing the proposed NTR selection algorithm for the given wireless network in Figure 2, node 10 and node 14 are NTR candidates because their maximum HCs are the same. However, since node 10 is larger than node 14 in terms of degree, node 10 is finally selected as the NTR node for the given wireless network in the proposed NTR selection. In the conventional NTR selection, the NTR node can be selected among node 1, having the largest UID, or node 20, having the smallest UID. In particular, when node 1 is selected as the NTR node, the time synchronization message is relayed to all nodes through maximum HC. Therefore, a long convergence time is required and the usage of time resources increases in this case. Furthermore, Figure 7b is shown as the average time error according to UIDs. As a result, when the selected NTR node is node 10, it achieves the best performance in terms of the average time error. When node 14, which is one of the NTR candidates, is selected as the NTR node, it achieves good average time error performance. However, if the NTR node is determined among node 1 or node 16, the HC required for time synchronization increases and time synchronization performance is significantly reduced.

**Figure 8:**
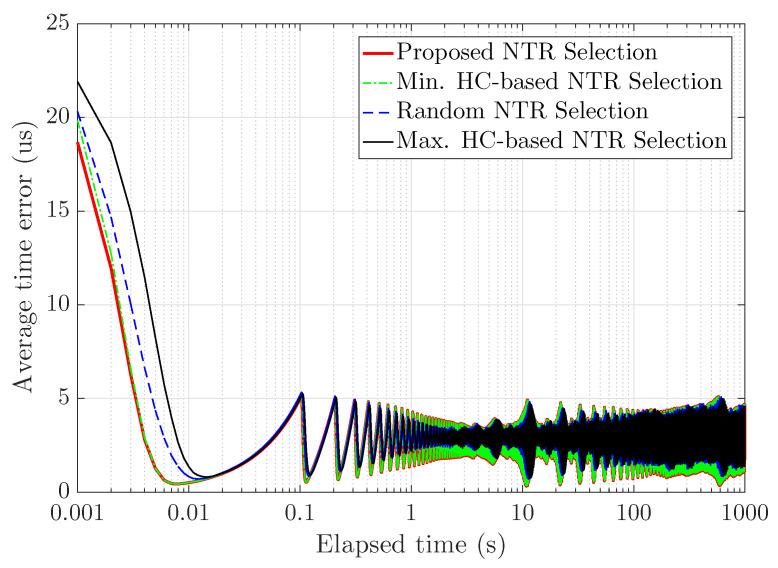
Average time error with respect to elapsed time.

By extending simulations, we evaluate performance of typical wireless ad hoc networks. Figure 8 shows the average time error with respect to elapsed time during 1000 [s] in 300 snap-shots of a wireless ad hoc network consisting of 100 nodes with 100 clock rates and initial clock offsets. Considering multiple snap-shots of a wireless ad hoc network, the average time error fluctuates because the HCs required for time synchronization are different from each other snap-shots. Note that the length of the time synchronization interval and HC required for time synchronization in each scheme are determined. As results, the proposed NTR selection achieves the shortest convergence time versus all baseline schemes. The minimum HC-based NTR selection achieves the same convergence time as the proposed NTR selection due to the same HC requirement for time synchronization, on the other hand, the random NTR seleciton and the maximum HC-based NTR selection have relatively long convergence time versus the proposed NTR selection and the minimum HC-based NTR selection. Therefore, the proposed NTR selcection can effectively reduce the convergence time and the average time error.

**Figure 9:**
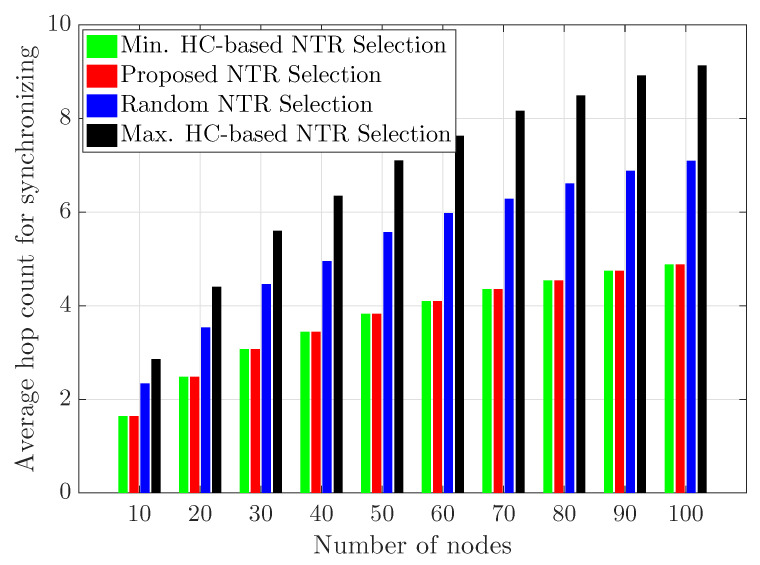
Average hop count for synchronization with respect to number of ad hoc nodes.

**Figure 10:**
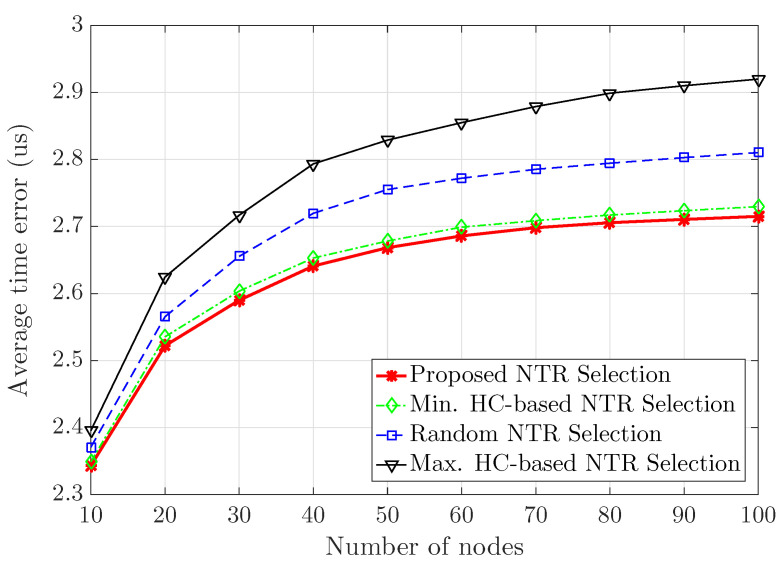
Average time error with respect to number of ad hoc nodes.

Figure 9 and Figure 10 show the average hop count for synchronizing and the average time error with respect to the number of ad hoc nodes from 10 to 100, respectively. Here, it should not be forgotten that all ad hoc nodes exist within the size of area 1000 [m] × 1000 [m]. Therefore, as the number of ad hoc nodes increases, the node density also increases. We consider that simulation time is equal to 100 [s] in 10,000 snap-shots of a wireless ad hoc network with 100 clock rates and initial clock offsets. Since the number of ad hoc nodes within a limited area increases, the density of the ad hoc nodes within the area increases, so the hop count for time synchronization does not increase linearly. In addition, when the number of ad hoc nodes is 100, the gap of the average hop count for synchronizing between NTR selections becomes larger. The proposed NTR selection and the minimum HC-based NTR selection have the same hop count required for the time synchronization of terminals in the wireless ad hoc network. These results show that the proposed NTR selection and the minimum HC-based NTR selection can efficiently use the time resource required for time synchronization. When the number of nodes is equal to 100, the proposed NTR selection, the minimum HC-based NTR selection, the random NTR selection and the maximum HC-based NTR selection achieve 2.715, 2.73, 2.81 and 2.92 [us] in terms of the average time error, respectively. Thus, the proposed NTR selection can achieve an improved average time error performance of about 0.55% compared to the minimum HC-based NTR selection, that of about 3.38% compared to the random NTR selection, and that of about 7.02% compared to the maximum HC-based NTR selection.

**Figure 11:**
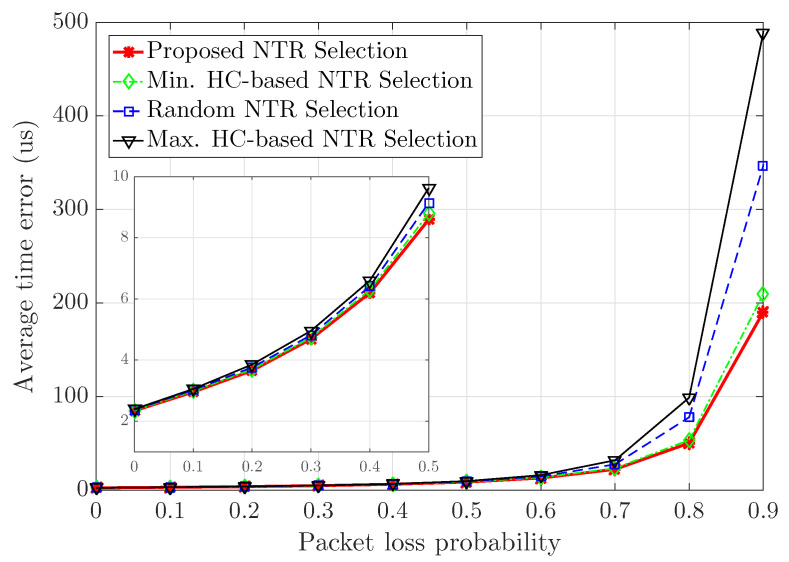
Average time error with respect to packet loss probability.

All of the previous results were analyzed in scenarios in which packet loss does not occur. Now, the following is the simulation results of the average time error performance when considering packet loss. In this paper, the *packet loss probability* is defined as the probability $P_{l}$ that a child node cannot receive any time synchronization message from its parent node because this message is lost when it relays the time synchronization message to its child node. Therefore, when $N_{p}$ parent nodes relay time synchronization messages in a certain subslot, the probability that a child node successfully receives this message becomes at least $\left(1-P_{l}^{N_{p}}\right)$. That is, when the number of parent nodes of a certain node is large, the probability that the node successfully receives at least one time synchronization message relayed from multiple parent nodes increases. Figure 11 shows the average time error with respect to packet loss probability from 0 to 0.9. We also consider that the simulation time is equal to 100 [s] in 10,000 snap-shots of a wireless ad hoc network consisting of 20 nodes with 100 clock rates and initial clock offsets. Obviously, the average time error increases as the packet loss probability increases. When the packet loss probability is equal to 0.9, the proposed NTR selection, the minimum HC-based NTR seleciton, the random NTR selection and the maximum HC-based NTR selection achieve 190.3, 209.8, 346.8 and 489.2 [us] in terms of the average time error, respectively. The proposed NTR selection can achieve an improved average time error performance of about 9.29% compared to the minimum HC-based NTR selection, that of about 45.13% compared to the random NTR selection, and that of about 61.10% compared to the maximum HC-based NTR selection. Therefore, the proposed NTR selection performs better in terms of average time error than all baselines due to HC-based and degree-based NTR selection considering packet loss occurance.

## 5. Conclusions

In this paper, we proposed a novel time synchronization protocol for time-division multiple access (TDMA)-based wireless barrage relay networks. The proposed time synchronization protocol operates based on cooperative barrage relay transmission in which nodes that have the same hop count from the network time reference (NTR) node in a cluster consisting of fully-connected ad hoc nodes. All nodes relay time synchronization messages containing the NTR node’s time-stamp and use this message to perform time synchronization. We also proposed a novel NTR selection technique for improving the convergence time and average time error. The proposed NTR selection can effectively reduce inefficiency that may occur when the NTR node exists at the end of the wireless ad hoc network. Extensive computer simulations show that the proposed protocol efficiently improves the time synchronization performance, compared with the conventional methods. The proposed techniques can be easily applied to practical distributed ad hoc networks where a global navigation satellite system (GNSS) service is not available or there is no central controller such as a base station or access point (AP). In order to improve the accuracy of time synchronization, it is important to perform time synchronization frequently. Thus, there is a fundamental trade-off between the time synchronization period and data transmission period. We leave the throughput, outage and latency in TDMA-based wireless barrage relay networks as further studies. 

## Figures and Tables

**Table 1:** Comparison with existing techniques in TDMA-based wireless ad hoc networks.

Reference	NTR Selection	Experiment/Simulation	Performance Metric
[25]	Fixed (central)	Experiment	Average/maximum time error
[26]	Fixed	Experiment	Maximum time error
[27]	*k*-medoids	Simulation	Average time error
[28]	Fixed	Experiment	Average time error

**Table 2:** Simulation parameters.

Parameters	Value
Simulation time	100[s] and 1000[s]
Size of area	1000[m]×1000[m]
Number of ad hoc nodes within a given area	from 10 to 100
Transmission range	100[m]
Length of subslot	0.001[s]
Time synchronization interval	0.1[s]
Clock skew	Uniform [−0.0001,+0.0001]
Clock rate	1+ Uniform [−0.0001,+0.0001]
Clock offset	Uniform [0,1]
Number of clock rates and clock offsets	100
Packet loss probability	from 0 to 0.9

## Data Availability

Data sharing not applicable.

## Abbreviations

The following abbreviations are used in this manuscript:

MAC	Medium access control
TDMA	Time-division multiple access
NTR	Network time reference
UID	User identifier
HC	Hop count
AP	Access point
CO	Clock oscillator
GNSS	Global navigation satellite system
GPS	Global positioning system
NLOS	Non line-of-sight
IEEE	Institute of electrical and electronics engineers
TSF	Time synchronization function
VANET	Vehicular ad hoc network
